# Implementing Cargo Movement into Climate Based Risk Assessment of Vector-Borne Diseases

**DOI:** 10.3390/ijerph110303360

**Published:** 2014-03-20

**Authors:** Stephanie Margarete Thomas, Nils Benjamin Tjaden, Sanne van den Bos, Carl Beierkuhnlein

**Affiliations:** Department of Biogeography, University of Bayreuth, Universitaetsstrasse 30, D-95447 Bayreuth, Germany; E-Mails: nils.tjaden@uni-bayreuth.de (N.B.T.); sanne.v.d.bos@web.de (S.B.); carl.beierkuhnlein@uni-bayreuth.de (C.B.)

**Keywords:** Asian tiger mosquito, global change, globalization, global warming, health hazards, invasive species

## Abstract

During the last decades the disease vector *Aedes albopictus* (Asian tiger mosquito) has rapidly spread around the globe. Global shipment of goods contributes to its permanent introduction. Invaded regions are facing novel and serious public health concerns, especially regarding the transmission of formerly non-endemic arboviruses such as dengue and chikungunya. The further development and potential spread to other regions depends largely on their climatic suitability. Here, we have developed a tool for identifying and prioritizing European areas at risk for the establishment of *Aedes albopictus* by taking into account, for the first time, the freight imports from this mosquito’s endemic countries and the climate suitability at harbors and their surrounding regions. In a second step we consider the further transport of containers by train and inland waterways because these types of transport can be well controlled. We identify European regions at risk, where a huge amount of transported goods meet climatically suitable conditions for the disease vector. The current and future suitability of the climate for *Aedes albopictus* was modeled by a correlative niche model approach and the Regional Climate Model COSMO-CLM. This risk assessment combines impacts of globalization and global warming to improve effective and proactive interventions in disease vector surveillance and control actions.

## 1. Introduction

The public perception of global change and biodiversity loss is dominated by climate warming, land cover changes, pollution, and non-sustainable over-exploitation of resources [1,2]. However, the increased connectivity between geographically remote continents via the transport of goods and travelling people is perhaps the most effective change that came into place during the last century. In many cases it is even the first time within evolutionary time scales that biotic exchange is enabled between formerly separated biota [3]. Regionally, and especially in urban areas, such an exchange and successful establishment of species that have evolved in other parts of the world may even lead to increased numbers of species. However, the missing regulation by food webs, as well as non-existent defense and adaptation strategies of local biota, will more likely contribute to intensified threats to native ecosystems and to the loss of ecological functioning and stability. 

The novel quality of anthropogenic transport systems with respect to the distances that are overcome, but also due to their speed and quantitative importance, is breath-taking compared to the situation just one century ago [4]. Nevertheless, travelling to far away holiday destinations and the consumption of goods that are produced in exotic countries, or the trade of industrial products across the oceans are generally perceived positively. The related biogeographically processes such as the spread of invasive species, pathogens and pests have first been realized on sensitive, because of long-term isolation and relatively poor species numbers, islands like New Zealand. Here, biosecurity and the control of incoming goods has been an issue for a long time [5]. 

In recent years however, novel qualities of global change are reinforcing the spread of invasive biota and pathogens. Changing temperature and modified precipitation regimes are opening new bioclimatic niches for the establishment of exotic species in many areas [6]. Additionally, temporally limited extreme conditions such as heat waves, drought periods or floods are creating windows of opportunity for the establishment of alien species [7]. As unintentionally dispersed arthropods that are capable of transmitting pathogens are increasingly appearing far outside of their historic natural ranges [8,9], their potential survival and establishment in those recently reached new areas, remain of great interest for vector-borne disease risk assessment. 

Travelers and tourists are carrying pathogens from endemic regions to non-endemic ones, in many cases not knowing about it when on the trip. As long as the target region does not exhibit competent arthropod vectors for the specific kind of disease, because these have been excluded by natural barriers or by the regional climate, this will stay ineffective and harmful for just the individual patient. However, there are two options for emerging and regionally novel vector-borne diseases: either the pathogen can be transmitted by local arthropod vectors and was just not introduced before, or competent vector species were recently introduced and established. 

Until today, a series of studies have addressed vector-borne diseases under changing climatic conditions both concerning climate trends and extreme events [10,11,12,13,14,15]. However, the starting point of a climate triggered establishment of vectors is the introduction that occurred [16]. It is thus important to integrate trade flow of goods in climate change based risk assessment of suitable regions for vector establishment and climate induced spread of vector-borne diseases.

Here, we focus on the role of anthropogenic transport (e.g., ships, trains) for the spread of competent vector species for diseases. Especially, we are interested in identifying the gateways, entry portals and hubs of exchange relevant for human health. Also we highlight the importance of one invasive insect, which has recently developed extensive populations outside its native range: *Aedes albopictus* (*Ae. albopictus*) better known as the Asian tiger mosquito.

This important vector for various arboviruses native to Southeast Asia has spread to all continents except Antarctica within the last four decades [17]. In this special case, the global shipping of goods such as used tires or ornamental plants like “lucky bamboo” contributes permanently to its introduction close to harbors [16]. When the climatic requirements of the species are met, establishment is highly likely if biosecurity is ignored [18].

Invaded regions are facing novel and serious public health concerns, especially regarding the transmission of formerly non-endemic arborviruses such as dengue and chikungunya [19,20]. In Europe, this mosquito has managed to establish permanent populations in large parts of the Mediterranean [21]. The further development and potential spread to other regions is largely dependent on their current and future climatic suitability [22] and the unintentional spread by transport and travel.

In this study, we identify and prioritize European areas at risk for the establishment of *Ae. albopictus* by taking into account, for the first time, the freight container imports from the mosquito’s endemic countries and the climate suitability of harbors and their surrounding regions. In a second step we consider the further transport of containers by train and by inland waterways within Europe, because these types of transport can be well controlled. The role of transport via roads and trucks must be addressed explicitly in future studies. This impact assessment aims to support effective and proactive interventions in disease vector surveillance and control actions.

## 2. Material and Methods

The study is based on data about the amount of cargo that is transported to and within Europe. As *Ae. albopictus* is proven to be transported by ship over large distances, harbors are the most important gateway of introduction. The amount of incoming cargo for the year 2011 at the top ten European harbors (Rotterdam, Hamburg, Antwerpen, Bremerhaven, Felixstowe, Valencia, Gioia Tauro, Le Havre, Algeciras and Barcelona) was obtained from the Eurostat database [23]. In the next step we determined for each harbor the proportion of incoming cargo from countries where *Ae. albopictus* occurs.

Freight data for European railways and inland-waterways were also taken from Eurostat. These data allow tracking of the amount of general cargo being moved from one European Region (NUTS2) to another. Road transport data was only available for far larger scales (national level NUTS0), so we were unable to include this important part of transport infrastructure in our analyses.

Outgoing cargo from the regions containing the harbors of interest to any other region in the EU was considered, as long as either the receiving, or the exporting nation had submitted data for this route (if both had, the mean was used; data provided by en-route-nations was discarded). The amount of outgoing cargo at each harbor to a specific European Region (NUTS2) was multiplied with the proportion of incoming cargo from endemic regions to total incoming cargo. Outgoing cargo from a NUTS2 region includes not only incoming cargo but also locally produced goods. However, we assumed the amount of locally produced goods to be negligible compared to the huge amount of incoming cargo at Europe’s top ten harbors. Incoming cargo for the specific receiving regions was summed up for railway and inland waterway bound cargo separately and divided by the size of the receiving region. ESRI ArcGis 10 was used to spatially analyze the received cargo per region in t/km².

Areas with a high probability of *Ae. albopictus* introduction, that in addition exhibit a high potential for the mosquito’s establishment, were identified by joining data and projections for infrastructure and climatic conditions. For this purpose we combined the spatial information on anthropogenic vectors (capacity of harbors, rail transport, and inland water transport) with a climate-based species distribution model (SDM).

We used a correlative bioclimatic niche model previously published by Fischer *et al.* (“global-driven statistic based model” from [24]) for the projection of the future regional fulfillment of climatic habitat requirements. The SDM was realized using the MaxEnt software for species distribution modeling [25] and built upon a global set of known points of the species’ distribution to reflect the whole range of known occupied niches [26,27]. The relevant bio-climatic variables (annual mean temperature, annual precipitation, precipitations of the warmest and coldest quarters as well as altitude; see [24] for details on the variable selection process) were obtained from the Bioclim dataset, available from worldclim.org. The mean AUC (area under the curve for the receiver-operator characteristic) value of 0.89 for 100 model runs with different, randomly selected test and training data subsets indicates a very good model fit [24].

Future projections on the European climatic suitability are based on the regional climate model COSMO-CLM [28]. While this prevented us from using the scenarios of the recently published final draft of Working Group I of the 5th Assessment of the IPCC [29], it bears the advantage of having a regional climate model adapted to the study area as to rely on the more general approach of a Global Circulation Model. Hence, our model is based on the climate projections for the A1B scenario of the 4th IPCC Assessment [30], which up to now has the highest likelihood of occurrence. For the combination with the amount of inbound cargo, the gridded suitability maps produced by MaxEnt with continuous values between 0 (low suitability) and 1 (high suitability) were divided into 7 classes. The threshold value for the climatic suitability (0.371 determined via equalization of sensitivity and specificity SeSpeql, see Fischer *et al.* [24]) is in the medium category, three categories are found below and three above this threshold.

The geographical vector datasets containing the information about the amount of inbound cargo were rasterized to match the format of the SDMs, using the “raster”-package [31] for R3.0.1 [32]. The continuous values of received cargo per area were accumulated into classes, keeping railroad (9 classes) and inland-waterway-related cargo (8 classes) separate, because of the loss of information that would have resulted from mixing the two, due to highly differing ranges of values. The class values of the SDM-raster were then added to the class values of the two cargo-rasters, creating two new grids of combined risk for each time frame (current climate, 2011–2040, 2041–2070, and 2071–2100, Table 1 and Table 2). Since to our knowledge no reliable, route-specific predictions for the development of cargo movement across Europe exist for the upcoming century, the amount of cargo was kept constant over all time frames.

**Table 1:** Combined risk evaluation taking into account climatic suitability for *Aedes albopictus* establishment and received cargo per area via inland waterway transport.

		Cargo via Inland Waterways (t per km^2^)							
		1	2	3	4	5	6	7	8
		(1–50)	(50–100)	(100–250)	(250–500)	(500–1,000)	(1,000–5,000)	(5,000–10,000)	(10,000–20,000)
**Climatic suitability for *Aedes albopictus* establishment (SDM)**	1 (0–0.1)	2	3	4	5	6	7	8	9
	2 (0.1–0.2)	3	4	5	6	7	8	9	10
	3 (0.2–0.3)	4	5	6	7	8	9	10	11
	4 (0.3–0.4)	5	6	7	8	9	10	11	12
	5 (0.4–0.5)	6	7	8	9	10	11	12	13
	6 (0.5–0.6)	7	8	9	10	11	12	13	14
	7 (0.6–1)	8	9	10	11	12	13	14	15

**Table 2:** Combined risk evaluation taking into account climatic suitability for *Aedes albopictus* establishment and received freight per area via railway transport.

		Freight via Train (t per km^2^)								
		1	2	3	4	5	6	7	8	9
		(1–10)	(10–20)	(20–40)	(40–60)	(60–80)	(80–100)	(100–500)	(500–1,000)	(1,000–3,000)
**Climatic suitability for *Aedes albopictus* establishment (SDM)**	1 (0–0.1)	2	3	4	5	6	7	8	9	10
	2 (0.1–0.2)	3	4	5	6	7	8	9	10	11
	3 (0.2–0.3)	4	5	6	7	8	9	10	11	12
	4 (0.3–0.4)	5	6	7	8	9	10	11	12	13
	5 (0.4–0.5)	6	7	8	9	10	11	12	13	14
	6 (0.5–0.6)	7	8	9	10	11	12	13	14	15
	7 (0.6–1)	8	9	10	11	12	13	14	15	16

## 3. Results

**Figure 1:**
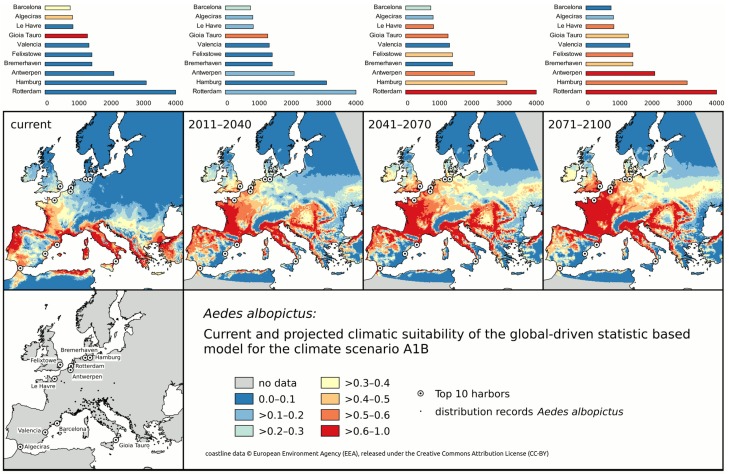
Changing climate suitability for *Aedes albopictus* establishment (adapted after [24], threshold value for the climatic suitability is 0.37 determined via equalization of sensitivity and specificity) during this century for the ten most important European harbors, arranged according to the amount of imported goods from endemic countries of *Aedes albopictus* (thousand tons).

The proportion of incoming cargo from *Ae. albopictus* endemic countries is highest in Rotterdam, Hamburg and Antwerp with up to 4 million TEU per year. Currently, these harbors located in the northern parts of central Europe exhibit no or only a very limited climatic suitability for *Ae. albopictus* establishment. However before the end of the century the situation is expected to change dramatically. Then these busiest harbors are projected to have optimal climatic conditions for Asian tiger mosquito’s establishment (Figure 1).

However most of the incoming goods at the harbors are moved further on, unopened, by ship, train or trucks. The probability of introduction of disease vectors by infested goods is thus shifted to the individual endpoints of the transport chain. 

### 3.1. Transport of Cargo by Inland Waterways

**Figure 2:**
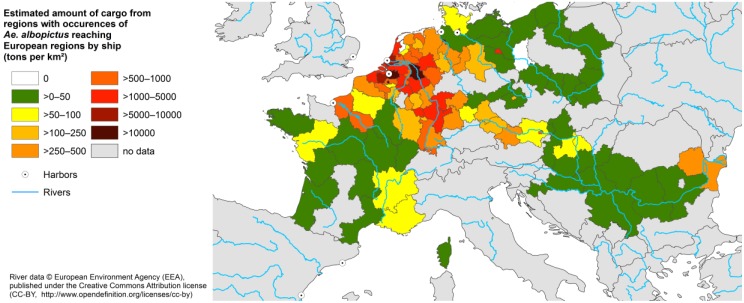
Transport of cargo from *Aedes* endemic countries from the top ten leading harbors by inland waterways to European NUTS2 regions (tons per km²).

Inland waterways are quantitatively the most important freight traffic system when compared to transport by rail, in regard to the further transporting of imports from *Aedes* endemic countries from the seaports onwards. The amount of cargo transferred by inland shipping reaches up to 10 times the amount of goods transported by train. These European NUTS2 regions adjacent to the main inland waterways which exhibit a high amount of unloaded cargo per area are highlighted (Figure 2). NUTS2 regions bordering at the Rhine, headwater area of Danube, Danube delta, Weser, Meuse and Seine show the highest transshipments (Figure 2).

**Figure 3:**
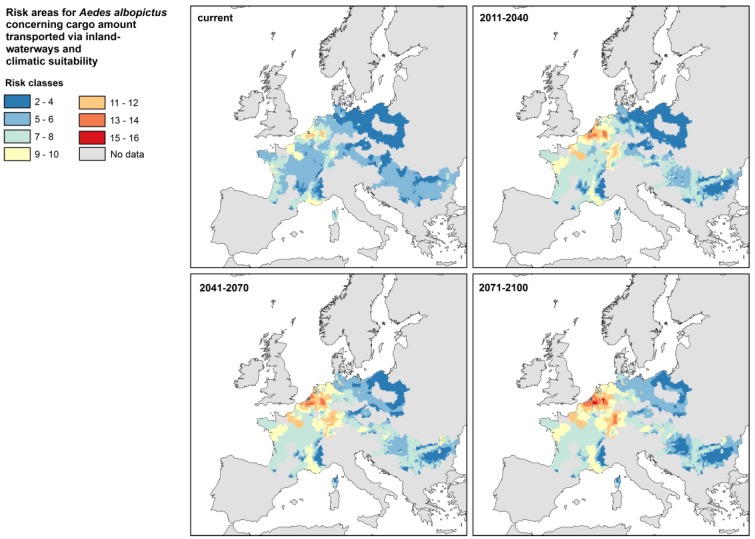
Risk for the establishment of *Aedes albopictus* by taking into account both, the amount of cargo imports from the mosquito’s endemic countries via harbors/inland waterways and the climate suitability of the recipient regions for mosquito’s long-term establishment.

The cargo amount from *Aedes* endemic countries transported by inland waterways is included into the risk assessment of the mosquito’s climate change driven establishment (Figure 3). 

The risk is currently most pronounced in densely populated areas, such as Le Havre and Paris (France), Ruhr area and Baden-Württemberg (Germany), Belgium, and the Netherlands. There, the risk substantially increases during the subsequent decades until the end of the century and additionally the area of high risk zones expands. Regions bordering the Danube exhibit a scattered pattern of different medium risk level zones, which are spatially shifting during this century.

### 3.2. Transport of Freight by Train

**Figure 4:**
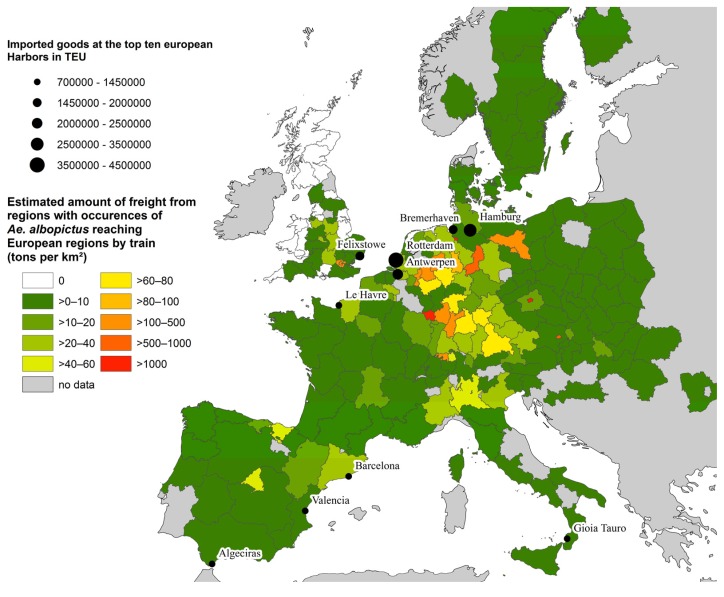
Transport of cargo from *Aedes* endemic countries from the top ten leading harbors by train to European NUTS2 regions (tons per km²).

Areas affected by imports from *Aedes* endemic countries further transported by rail are focused mainly on central Europe. These are in particular the Benelux states, Germany, Switzerland, Austria, Northeastern Spain (Catalonia and Aragon) and North Italy (Po Valley) (Figure 4). Moreover, a small number of clearly geographically defined areas around metropolises such as London, Prague and Vienna show high incoming amounts of rail-bounded freight. 

**Figure 5:**
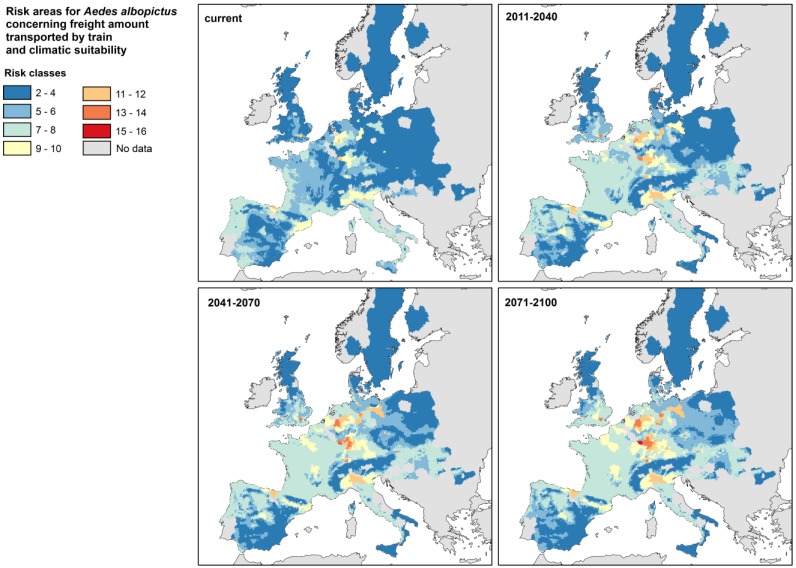
Risk for the establishment of *Aedes albopictus* by taking into account both, the amount of freight imports from the mosquito’s endemic countries via harbors/railways and the climate suitability of the recipient regions for mosquito’s long-term establishment.

Combining the risk of introduction by train freight and the suitability of climatic conditions for long term establishment of the Asian tiger mosquito, the strongest change in the distribution and assignment to risk classes is found during the coming three decades (Figure 5). From then on changes are marginal and primarily involve zones already at high risk; here there will be a further intensification of risk classification.

The amount of goods transported by train is three times as high as the amount of goods transported by inland waterways, and not spatially limited to large navigable river systems. General tendencies of both risk assessments are similar: the highest risk classes are found in central Europe (Benelux states, Germany, northern France). There, risk is increasing until the mid of the century and remains at a high level thereafter.

## 4. Discussion

Global climatic changes are causing the spread of invasive species on all continents [6,33]. But, in most cases the initial establishment of alien species is caused by human trade and transport of goods [34]. Global markets have become closely connected; harbors and airports are hotspots of import and turnover. But still, effective biocontrol is limited to sensitive places such as islands. Continental hubs of import such as large harbors or freight terminals are less monitored. But there are first signs of alert also at the European scale [35], as such, invasive species are increasingly perceived as a complex threat for continental Europe [36]. In this study, we combine climatic projections and species distribution models, with geo-information on human infrastructure and freight distribution by transport at the European scale. In the light of our results, we can see that results from previous studies (e.g., [24,37]) have yielded patterns of spatio-temporal spread of the species that were far too regular, as they could not consider preferential dispersal.

When living specimens or viable propagules of invasive insects are reaching a new area outside of their previously natural range of distribution, it is decisive whether these points of intersection and transportation nodes can be controlled. If not, they are likely to serve as efficient hubs for the rapid distribution of these problematic organisms via anthropogenic vectors, such as the transport of goods on roads, railways or ships. Our study is aiming to attract attention to these important lines of connectivity.

The body size, abundance and behavior of invasive insects but also their life-cycles and breeding habits of invasive insects are particularly suitable for passive transport by means of human vehicles and freight containers. In the case of invasive forest insect pests, sustained damage has already stimulated sensitivity and safety precautions [38]. Astonishingly, this is not yet the case for disease vectors although there is incidence for novel establishments (e.g., [39,40]).

As a natural infrastructure for transport by ship, large rivers show a specific quality. Evidently, they are always located in the lowest and warmest parts of landscapes and along their valleys, climatic conditions change very gradually. This makes rivers and valleys biodiversity hotlines [41]. We show in this European case study that the preferential topography of this means of transport is directing the freight along corridors of high climatic suitability for disease vector establishment.

In contrast to large rivers and valleys, roads and railroads are crossing a variety of landscapes and climatic conditions also leading to the passive transportation of “carry-on organisms” into areas where they are unfit for survival. In view of the large numbers of transported specimens, such tremendous sinks of their meta-population can be tolerated. However, within a few hours, vehicles that are inadvertently carrying alien species are also reaching fragmented and isolated areas, which exhibit suitable conditions for the establishment and regional spread of insect vectors. Our study confirms that areas that have been segregated from other parts of the mainland by efficient natural barriers, such as mountain ranges, lakes or rivers are likely to be reached. In consequence, various ecological niches are not occupied and biotic interaction, such as predation, are less developed. Under these conditions, invasive species have high prospects of success. During the last two years, live tiger mosquitos have been recorded close to motorways north of the Alps (e.g., [42]). However, as long as their eggs do not survive low temperatures during winter, it is unlikely that this species will generate viable populations in the landscape [43]. 

Urban areas are of high importance in risk assessment, as cargo is transported mainly towards a small range of urban destinations. Colunga-Garcia *et al.* [38] calculate, for the transport of wood products, that 84%–88 % of the imported tonnage went to only 4%–8% of the United States urban areas. As urban areas are already much warmer than their surrounding landscapes, it is very likely that they will be the hot spots of initial establishment for thermophilic disease vectors. The density of the human population and the risk of unaware pathogen infections with incoming travelers could precipitate a critical situation if a competent vector is able to establish. This is why a combined approach is required that considers the climatic constraints of both, the vector and the pathogen [44].

Species distribution models, as they are used in this study, have become a powerful tool in research on the ecological consequences of climate change in general [45] but also for insect vectors such as *Ae. albopictus* (e.g., [22,24]). Today, models are becoming more realistic by integrating insect species traits, such as the specific dispersal ability [46,47]. But for most insect vectors our knowledge is scarce, and of course, there is no perfect model. Mechanistic and correlative models are just highlighting the problem from different angles [22]. It is evident that multi-disciplinary approaches are needed to address the upcoming risks in connection with invasive disease vectors in Europe [48].

All data on the intra-European transport used in this study refer to NUTS2-regions. A distinction between cargo that has arrived at and is being distributed from the local harbor and goods that have been produced within the region itself cannot be made. Hence, the larger a NUTS2-region close to one harbor is, the more uncertainty exists regarding the provenance of goods exported out of it. We identified the proportion of cargo coming from regions within the global distribution of *Ae. albopictus* for each European harbor. In this way, we estimate the risks connected to the inland transport coming from individual European harbors. However, data are not available to select certain types of goods that are preferably associated with the spread of *Ae. albopictus* (for instance used tires and “lucky bamboo”) and to track their distribution.

The results of this study are limited by the fact that qualitative and quantitative data on transport via roads could not be accessed at the required spatial resolution (NUTS2 level data for both source- and destination-regions). Similar data of broader resolution (country level for source- and NUTS2 level for destination-regions) does exist, but only small parts of it are publicly available at Eurostat while the major part is classified as “confidential”. Consequently, there is the main branch of intra EU transport missing. Nevertheless the approach presented here indicates how to identify and prioritize European hotspots for vector’s establishment by taking into account freight transport by train and inland waterways and the climate suitability. 

Apart from harbors, there are of course other potential sources for introduction. Airports are omitted from this study for the simple reason that they usually have no connection to inland waterway transport systems, and while they are often connected to railway systems, these air-rail links are seldom used for freight but rather passenger transport [49]. Air freight amounts to less than 0.4% of the cargo handled at harbors and includes also a large proportion of mail transport which could not be separated from freight transport. Also, the distribution of already established populations of *Ae. albopictus* in Europe was not considered in this pilot study. In the future, this could be done with small modifications to the workflow presented here, with missing data for road transport remaining the main obstacle.

## 5. Conclusions

Anthropogenic infrastructure and transport cannot be ignored when modeling the potential spread of disease vectors. Especially the gates of import and the networks of distribution must be considered. The physical habitat requirements of invasive arthropod disease vectors are often quite simple. In case of the Asian tiger mosquito (*Ae. albopictus*) any kind of freshwater body and even small volumes suffice for larval and pupae development. This indicates that anthropogenic introduction and passive long-distance transport of such a potentially harmful insect must be avoided. In this study we make a first step to improve species distribution models for these vectors. This will help to conduct efficient monitoring activities.
